# *Fusobacterium nucleatum* predicts a high risk of metastasis for esophageal squamous cell carcinoma

**DOI:** 10.1186/s12866-021-02352-6

**Published:** 2021-10-30

**Authors:** Zhen Li, Chao Shi, Jiawen Zheng, Yongjun Guo, Taibing Fan, Huan Zhao, Dongdong Jian, Xiaolei Cheng, Hao Tang, Jie Ma

**Affiliations:** 1 National Health Commission Key Laboratory of Cardiovascular Regenerative Medicine, Heart Center of Henan Provincial People’s Hospital, Central China Fuwai Hospital of Zhengzhou University, Central China Fuwai Hospital & Central China Branch of National Center for Cardiovascular Diseases, No.1 Fuwai Road, Henan province 450003 Zhengzhou, People’s Republic of China; 2 Microbiome Laboratory, Henan Provincial People’s Hospital, People’s Hospital of Zhengzhou University, Zhengzhou, 450003 Henan China; 3 Department of Molecular Pathology, The Affiliated Cancer Hospital of Zhengzhou University, Henan Cancer Hospital, No.127 Dongming Road, Henan province 450008 Zhengzhou, People’s Republic of China; 4 Henan Key Laboratory of Molecular Pathology, Zhengzhou, 450008 Henan China; 5 Department of Oncology, The first affiliated hospital of Zhengzhou University, Zhengzhou, 450052 Henan China

**Keywords:** ESCC, *Fusobacterium nucleatum*, Tumor mutation burden, Tumor progression, EGF (epidermal growth factor)

## Abstract

**Background:**

Esophageal squamous cell carcinoma (ESCC) is the major type of esophageal cancer in China. The role of the bacteria present in ESCC tissue in neoplastic progression has not been fully elucidated. This study aimed to uncover different bacterial communities in ESCC tissues and examine the correlation between the abundance of the esophageal flora and clinicopathologic characteristics of ESCC.

**Results:**

Microorganisms in tumors and normal tissues showed obvious clustering characteristics. The abundance of *Fusobacterium* (*P* = 0.0052) was increased in tumor tissues. The high level of *Fusobacterium nucleatum* was significantly associated with pT stage (*P* = 0.039) and clinical stage (*P* = 0.0039). The WES data showed that *COL22A1, TRBV10–1, CSMD3, SCN7A* and *PSG11* were present in only the *F. nucleatum*-positive ESCC samples. GO and protein domain enrichment results suggested that epidermal growth factor might be involved in the regulation of cell apoptosis in *F. nucleatum*-positive ESCC. Both a higher mutational burden and *F. nucleatum*-positive was observed in tumors with metastasis than in tumors without metastasis.

**Conclusion:**

*F. nucleatum* is closely related to the pT stage and clinical stage of ESCC. The abundance of *F. nucleatum* and tumor mutation burden may be used in combination as a potential method to predict metastasis in ESCC.

**Supplementary Information:**

The online version contains supplementary material available at 10.1186/s12866-021-02352-6.

## Background

Esophageal cancer is the main malignant tumor of the upper digestive tract worldwide. There are approximately 570 thousand new cases of esophageal cancer globally every year, of which more than 80% are new cases in developing countries, half of which are in China [1]. Esophageal squamous cell carcinoma (ESCC) is the major type of esophageal cancer in China, in contrast to western countries. Henan Province is a typical region with a high incidence and mortality of esophageal cancer in China. Despite the development of multimodal therapies, including surgery and chemotherapy radiotherapy, the prognosis of patients, including those who undergo complete resection, remains poor. The five-year survival rate of this disease has remained only approximately 10–25% [2]. Therefore, further studies are needed to clarify the pathogenesis of esophageal cancer and to explore new diagnostic and therapeutic possibilities.

Many studies have demonstrated that the composition of the microbiota in human skin and various mucous membranes (e.g., mouth, upper respiratory tract, lower genitourinary tract, gastrointestinal tract) is closely related to health and disease [3]. Digestive tract microorganisms are very important in all parts of the human body because they can directly or indirectly regulate the digestive system, immune system, nervous system, circulatory system, brain and other organ functions. In recent years, reports have paid attention to the microbiome as the key player triggering tumorigenesis. ***Helicobacter pylori*** infection is the strongest known risk factor for gastric adenocarcinoma [4], and it is also a risk factor for liver cancer, esophageal cancer and colorectal cancer; ***Fusobacterium nucleatum*** is the main cause of colorectal cancer and pancreatic cancer [5, 6]; ***Porphyromonas gingivalis*** has been proven to be closely related to the occurrence of oral squamous cell cancer, gastrointestinal cancer or pancreatic cancer [7]; and *Enterobacter*, *Bacteroides* and *Enterococcus* are also responsible for multiple organ cancers throughout the body. Recently, a microbiome analysis of seven human tumor microenvironments showed that bacteria are widely found in cancer cells and immune cells in various tumors, and the bacterial content is related to the type of tumor [8]. The crucial event in carcinogenesis triggered by the microbiome seems to be chronic inflammation influencing the genomic stability of host cells and activating immune mechanisms [9, 10].

Due to the limitation of sampling methods and esophageal environmental dynamics, the study of the microbiome in the esophagus is still in its infancy; in particular, the relationship between ESCC and the microflora needs to be further studied. Recent studies have shown that the diversity of the microflora in esophageal cancer tissues is significantly different from that in normal esophageal tissues [11] and it is associated with prognostic survival. ***F. nucleatum*** in esophageal cancer tissues is associated with shorter survival, suggesting a potential role as a prognostic biomarker [12, 13]. ***F. nucleatum*** might also contribute to aggressive tumor behavior through activation of chemokines, such as CCL20 [12]. However, little information is available concerning the association between microorganisms and pathological characteristics in ESCC.

In this study, 41 pairs of tumors and normal tissues of ESCC patients were used for 16S rRNA or whole-exome sequencing (WES) sequencing. We also quantified *F. nucleatum* DNA in 98 samples of ESCC by qPCR and detected the relationship between *F. nucleatum*, tumor gene mutations and clinical characteristics of ESCC**.** Moreover, the combined mutational burden and the content of *F. nucleatum* in tumors could predict postoperative tumor metastasis in ESCC. In brief, we focused on the relationship between the specific microbiome and pathological characteristics and explored its application prospects as a biomarker for the pathogenesis and progression of ESCC.

## Results

### Sample collection and clinical information

**Fig. 1:**
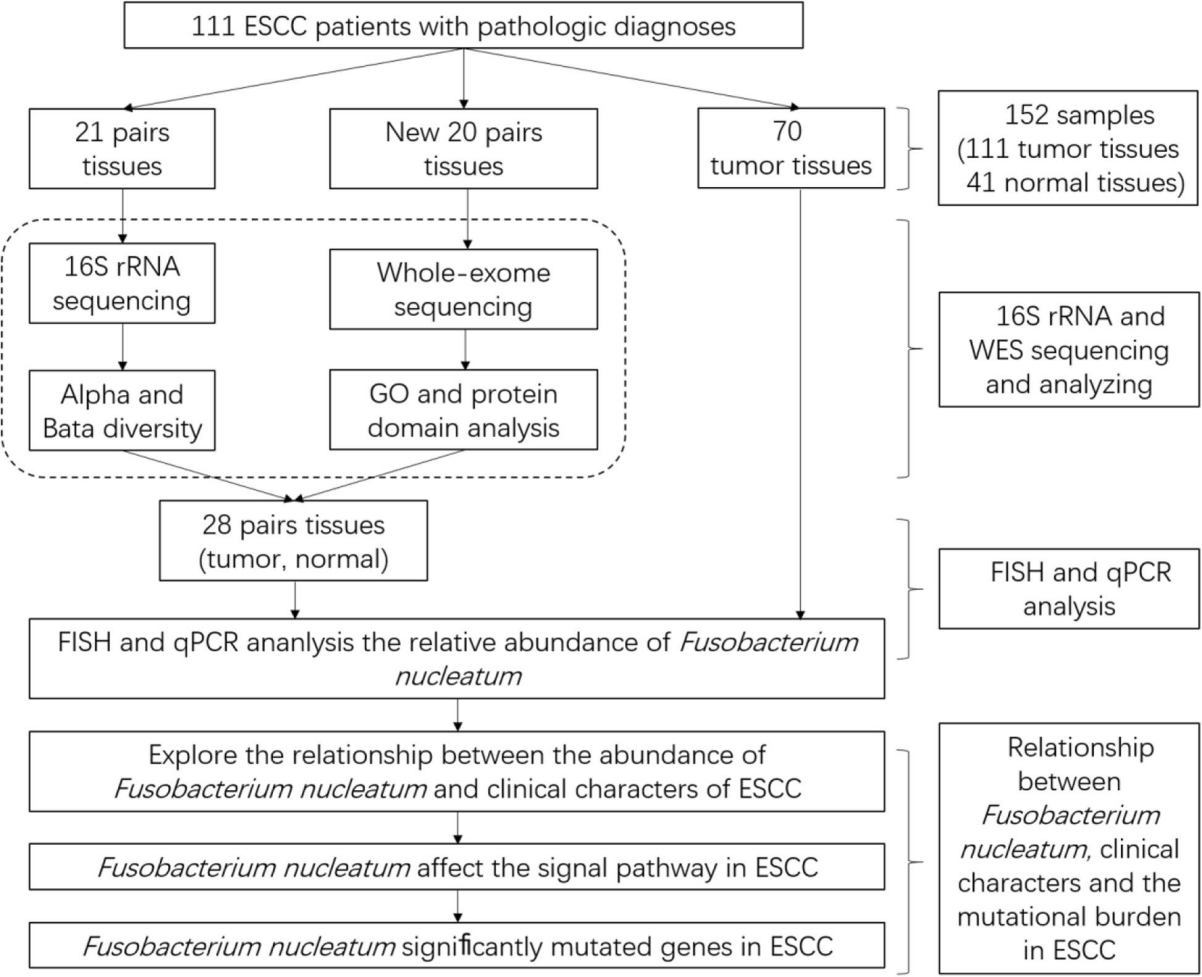
Diagram of study enrollment and eligible paired and total samples. ESCC indicates esophageal squamous cell carcinoma

In this study, we collected esophageal tissue wax blocks from 111 individuals, of which 42 included tumors and paired nontumor tissues and the other 69 were only tumor tissues (Fig. 1). Forty-one patients with ESCC were included, and microbiome samples from both tumor and normal tissues were collected. The clinical information of the 111 patients is shown in Table 1.

**Table 1:** Patient clinical characteristics and detection level of *F. nucleatum*

Clinical Characteristic	Total Number of Patients	Total Detect Number	Samples for detect ***F. nucleatum*** (***N*** = 98)		
			Positive	Negative	***P***-value
	111	98	68(69.4%)	30(29.6%)	
Age (mean, range)					
62.9 ± 7.1					
< 60	31(27.9%)	29	22(75.9%)	7(24.1%)	0.1412
> 60	80(72.7%)	69	46(66.7%)	23(33.3%)	
Gender					
Male	64(57.7%)	56	39(69.6%)	17(30.4%)	0.9989
Female	47(42.3%)	42	29(69%)	13(31%)	
Smoking					
Yes	44(39.6%)	39	26(66.7%)	13(33.3%)	0.9428
No	67(60.4%)	59	42(71.2%)	17(28.8%)	
Drinking					
Yes	46(41.4%)	39	25(64.1%)	14(35.9%)	0.5561
No	65(58.6%)	59	43(72.9%)	16(28.8%)	
Histopathological grading					
G1–2	79(71.2%)	72	50(69.4%)	22(30.6%)	0.5305
G3	32(28.8%)	26	18(69.2%)	8(30.8%)	
clinical T stage (pT)					
pT1–2	44(39.6%)	35	24(68.6%)	11(31.4%)	0.039
pT3–4	67(60.4%)	63	44(69.8%)	19(30.2%)	
lymph node					
pN0–1	97(87.4%)	89	61(68.5%)	28(31.5%)	0.1343
pN2–3	14(12.6%)	9	7(77.8%)	2(22.2%)	
Clinical tumor stage					
I	8(7.2%)	4	3(75%)	1(25%)	0.0033
II	71(64%)	68	44(64.7%)	24(35.3%)	
III	32(28.8%)	26	21(84%)	5(15.4%)	

### Microbiota diversity in ESCC

**Fig. 2:**
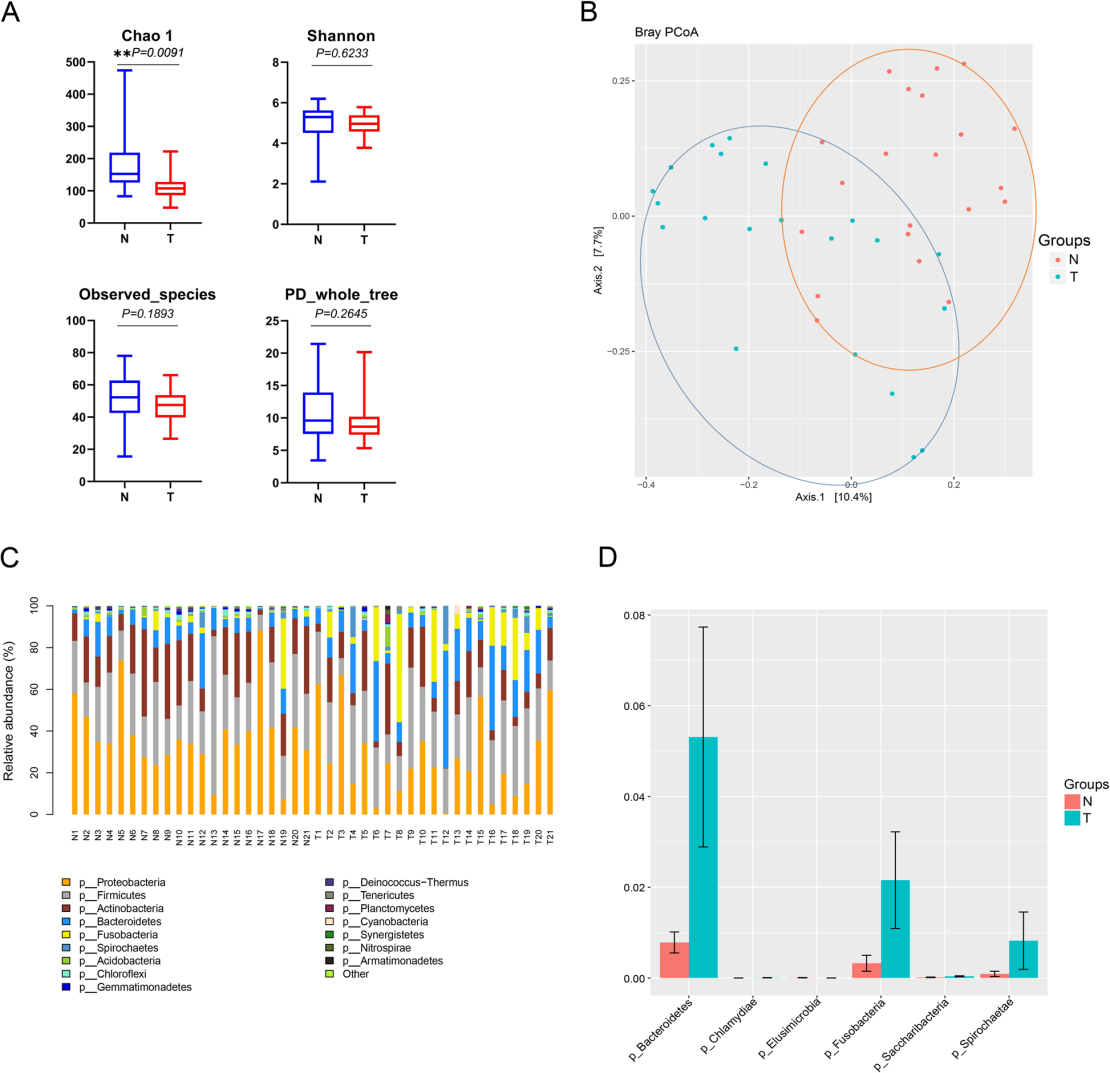
Microbial comparison of paired tumor and paired-normal tissues from patients with ESCC for α (**A**) and β diversity (**B**). The α diversity of 21 paired ESCC tumor and nontumor tissues was assessed with the Chao1 index, Shannon index, observed species and PD_whole_tree. The β diversity, based on the bray-curtis distance, was compared with PCoA plots for 21 paired tumor and nontumor tissues from patients with ESCC. PCoA, principal coordinate analysis. (**C**) The relative abundance of bacteria at the phylum level in both the normal and tumor groups of ESCC. (**D**) Differential abundance analysis revealed that *Bacteroidetes* (*P* = 0.03149), *Fusobacteria* (*P* = 0.01796) and *Spirochaetae* (*P* = 0.004155) were the top 3 significantly enriched taxa in the tumor group compared with the matched normal group samples. **P-value< 0.01

We generated 13,671,987 quality-filtered sequence reads, with an average of 48,311 reads per sample (Fig. S1A). Sequence reads were mapped to the bacteria in the SILVA database. In general, the 16S rRNA gene sequencing results of paired tumor and normal tissue microbial profiles show partial differences. For alpha-diversity, the Shannon index (4.93 vs 5.05, *P =* 0.6233), PD_whole_tree (9.35 vs 10.73, *P =* 0.2645) and the observed species (46.97 vs 52.29, *P =* 0.1893) were lower in the ESCC tumor tissues than in the normal tissues, but these differences were not statistically significant; however, the overall alpha-diversity Chao 1 index (132.06 vs 185.86, *P =* 0.0091) in tumor tissues decreased dramatically by approximately 25% compared with that in the matched normal tissues (Fig. 2A). Differences in microbial community structure between normal and tumor tissue were observed in ESCC patients using principal coordinate analysis (PCoA) ordination of the bray-curtis distance (beta-diversity). The PCoA plots showed that (Fig. 2B). Although there is a small overlap between the microbial composition of the normal and tumor tissues, the overall microbial composition remained different.

### Relative abundances of bacteria at the phylum level in both the normal and tumor groups of ESCC patients

To further determine the signature of microbial profiles in both the normal and tumor groups, the relative abundances of bacteria at the phylum level were determined, and the results are shown in Fig. 2C (Class level was shown in Fig. S1B). The top 6 phyla across all samples were *Actinobacteria*, *Bacteroidetes*, *Firmicutes*, *Fusobacteria*, *Proteobacteria* and *Spirochaetes*. Differential abundance analysis revealed a similar conclusion*: Bacteroidetes* (*P =* 0.03149*), Fusobacteria* (*P =* 0.01796*)* and *Spirochaetae* (*P =* 0.004155) were the top 3 significantly enriched taxa in the tumor group compared with the matched normal group samples (Fig. 2D). To identify the signature microbiota existing in tumor tissues, which potentially play roles during the carcinogenesis of ESCC, 16S rRNA sequencing data were annotated at the genus level. *Butyrivibrio* (*P =* 0.0078) and *Lactobacillus* (*P =* 0.00052) were closely associated with sugar and fiber fermentation and were found at significantly lower relative abundances in the tumor group than in the normal group. *Streptococcus* (*P =* 0.0013) was discovered increased in tumor group. In addition, *Fusobacterium* is anaerobic, gram-negative bacteria and normally treated as pathogen, which discovered as increasing relative abundance in tumor groups (*P =* 0.0052) (Fig. S2). The full list of signature microbiota profiles (genus level) is summarized in Table S1.

### Microbiota associated with clinical characters in ESCC

**Fig. 3:**
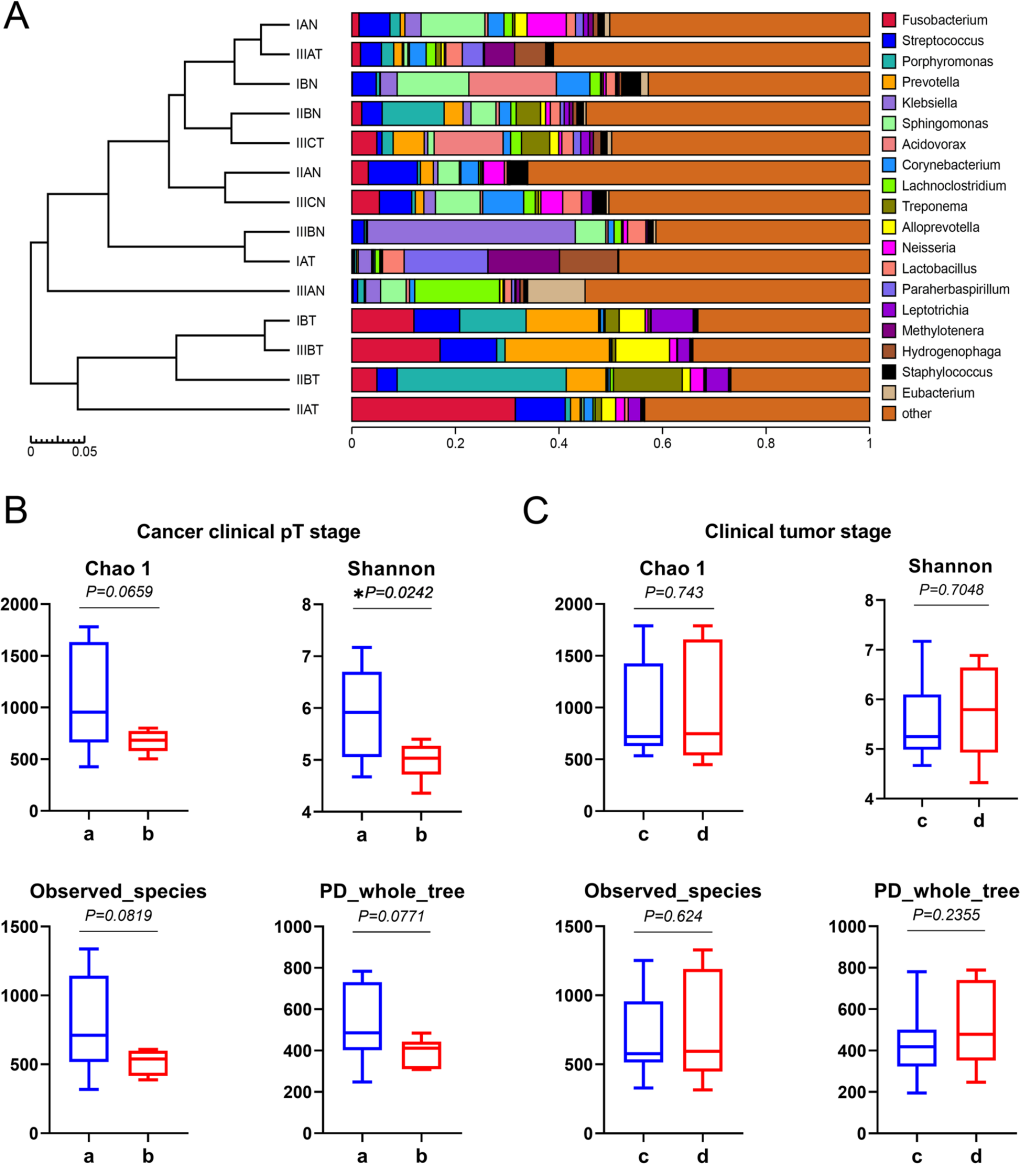
Microbiota associated with clinical tumor stage in ESCC. (**A**) Twenty-one pairs of samples according to their respective clinical stages and clustered trees based on the unweighted UniFrac distance matrix and UPGMA method; the clustering results were integrated with the relative abundance of each sample species. The α diversity at the clinical pT stage (**B**) and clinical tumor stage (**C**) was assessed with the Chao1 index, Shannon index, observed species and PD_whole_tree

We grouped 21 pairs samples according to their respective clinical stages. To explore whether there are regular differences among the compositions of microorganisms in ESCC, we clustered samples based on the relative abundances of each sample species, and used the unweighted UniFrac distance matrix and UPGMA method. The results of the histogram (Fig. 3A) and heatmap showed that the relative abundances of *Fusobacterium* (*P =* 0.039) and *Prevotella* (*P =* 0.0379) were correlated with clinical stage in ESCC, where they were higher in tumors than in the corresponding normal tissues (Fig. S3), not with the IA stage.

To further explore the microbiome alterations that occur during the progression of esophageal cancer and identify signature species as diagnostic biomarkers, we performed differential abundance analyses on 16S rRNA sequencing data of tumor tissues only using DEseq2, and cross-referenced with both patient clinical tumor classification of malignant tumor.

Subjects in the pT1-T2 classes were designated Group a, and those in pT3-T4 were designated Group b. In the pT classification, α diversity measures indicated a decreased diversity in Group b (Fig. 3B). Although there was no significant difference in the Chao1 (1094.03 vs 843.77, *P =* 0.0659) index, PD_whole_tree (493.81 vs 450.37, *P =* 0.0771) and the observed species (790.09 vs 626.27, *P =* 0.0819), the Shannon index (5.858 vs 5.219, *P =* 0.0242) decreased significantly in Group b. We found that the relative abundances of the phyla *Fusobacteria* (*P =* 0.0048) and *Bacteroidetes* (*P =* 0.0035) increased significantly in Group b (Fig. S4). In addition, multiple comparisons between every pT stage were performed, and bacterial taxa with significant changes in abundance are summarized in Table S2. Many bacterial genera were significantly changed, including *Lactobacillus*, members of which show preventive effects in bacterial infection and inflammation [14]. Moreover, data from clinical stage I-II subjects were grouped (Group c) and then compared with stage III subject tumor microbiome profiles (Group d). As illustrated in the figure (Fig. 3C), there were no statistically significant differences between groups c and d in α diversity as measured by the Chao1 index (1011.25 vs 984.73, *P* = 0.743), Shannon index (5.67 vs 5.64, *P* = 0.7048), PD_whole_tree (480.52 vs 464.56, *P* = 0.2355) and the observed species (741.26 vs 720.91, *P* = 0.624). The data showed that the community diversity and richness indices of microbiome were no statistically significant differences between stage I-II and stage III. In addition, the current analysis shows that the distribution of microflora diversity in tumor tissue is not related to the patient’s age, gender, smoking history, drinking history, tumor location, lymph node metastasis or G stage (Table S3).

### FISH detection

**Fig. 4:**
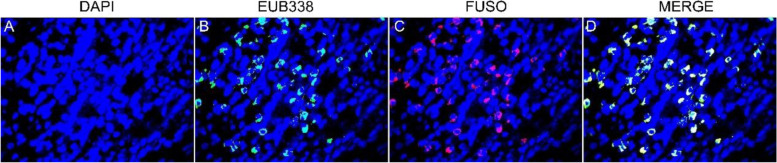
Enrichment of *Fusobacterium nucleatum* in ESCC detected by FISH. Graph **A**: ESCC specimens stained with DAPI. Cell nuclei were stained in blue; **B**: ESCC specimens stained with both DAPI and a universal bacterial probe (EUB338). Bacterial conserved regions were stained in green; **C**: ESCC specimen stained with both DAPI and a *Fusobacterium*-specific probe (FUSO). The *F. nucleatum*-specific regions were stained in red; **D**: ESCC specimens triply stained with DAPI, FUSO and EUB338. Cell nuclei (in blue), bacterial conserved regions (in green) and *F. nucleatum*-specific regions (in red) were clearly visible (all × 400)

*F. nucleatum* was detected in 20 specimens of ESCC tissue by FISH. *F. nucleatum* stained with the FUSO probe (in red) was found to be enriched within the esophageal tumor tissue (median, 23; range, 5 to 69) (Fig. 4).

### qPCR to verify the level of *F. nucleatum* in ESCC

**Fig. 5:**
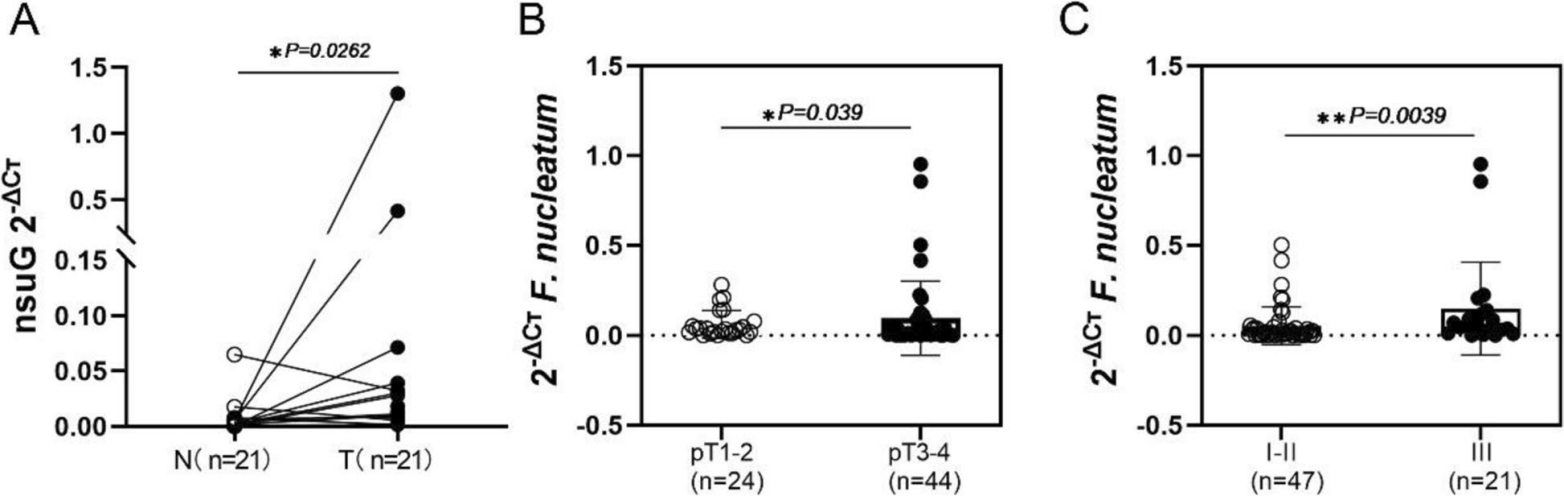
Distribution of *F. nucleatum* in ESCC. (**A**) The relative abundance of *F. nucleatum* in tumor tissues was significantly higher than that in paired normal tissue samples in 21 patients with ESCC (*P* = 0.0262). (**B**) The relative *F. nucleatum* levels in the pT3–4 stage were significantly higher than those in the pT1–2 stage (*P* = 0.039), and the enrichment was higher in the III stage than in the I-II stage (*P* = 0.0039)

To verify the results of 16S rRNA gene sequencing, we collected more samples to check the relationship between *F. nucleatum* and clinical characteristics of ESCC. To evaluate the relative abundance of *F. nucleatum* in tumor tissues, the specific *nusG* gene of *F. nucleatum* was quantitatively analyzed by qPCR in samples from 98 ESCC patients. The results showed that of the 98 tumor tissue samples, 69.4% (68/98) were positive for *F. nucleatum*, and the relative abundance of *F. nucleatum* in tumor tissues was significantly higher than that in paired normal tissues (*P* = 0.0262) (Fig. 5A). By univariate analysis, it was found that the abundance of *F. nucleatum* was highly correlated with the pT and clinical stages. The relative *F. nucleatum* levels in the pT3–4 stage were significantly higher than those in the pT1–2 stage (*P* = 0.039), and there was greater enrichment in the III stage than in the I-II stage (*P* = 0.0039) (Fig. 5B and C). This is consistent with the above analysis results showing that the relative *F. nucleatum* DNA levels in ESCC tumor tissues were not significantly changed patient age, gender, smoking history, drinking history, lymph node metastasis and G stage (Table 1 and Fig. S5).

### Mechanism by which *F. nucleatum* affects the progression of ESCC

**Fig. 6:**
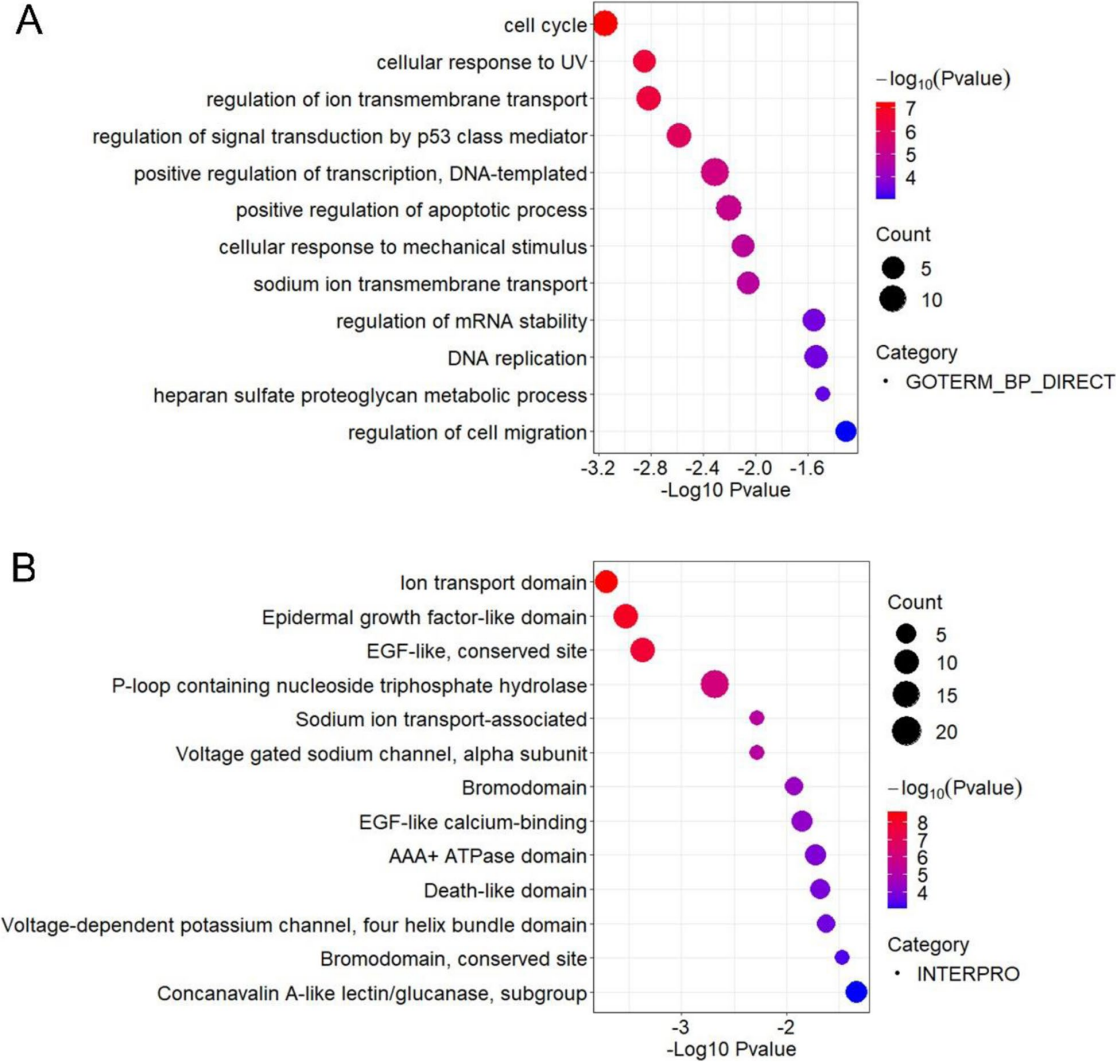
(**A**) Gene Ontology (GO) functional and (**B**) protein domain enrichment analysis of all the high-risk mutant genes of the *F. nucleatum*-positive samples

According to the qPCR results, we selected 20 pairs of ESCC samples for whole-exome sequencing (WES). ESCC samples were divided into two groups according to the abundance of *F. nucleatum*. Any sample with a Ct less than 37 was considered *F. nucleatum*-positive. Specimens were considered negative when the Ct value of the specimen was greater than 37 or no melt curve could be generated. Thirteen tumor tissues contained *F. nucleatum,* and the other 7 were *F. nucleatum* negative. GO functional enrichment analysis of all the high-risk mutant genes of the 13 *F. nucleatum*-positive samples was conducted, and the most significantly enriched functions are shown in Fig. 6A. The most significantly enriched functions included cell cycle (*P* = 0.00071), positive regulation of apoptotic process (*P* = 0.0063) and positive regulation of transcription, DNA-templated (*P* = 0.0049). Moreover, the involved proteins were further classified by protein domain, and it was found that the epidermal growth factor-related domain (EGF-like domain) was significantly enriched (*P* = 0.00029) (Fig. 6B). The enriched GO terms for high-risk mutant genes in *F. nucleatum*-positive specimens were significantly different from those for the 7 *F. nucleatum*-negative samples (Fig. S6).

### *F. nucleatum* significantly mutated genes in ESCC

**Fig. 7:**
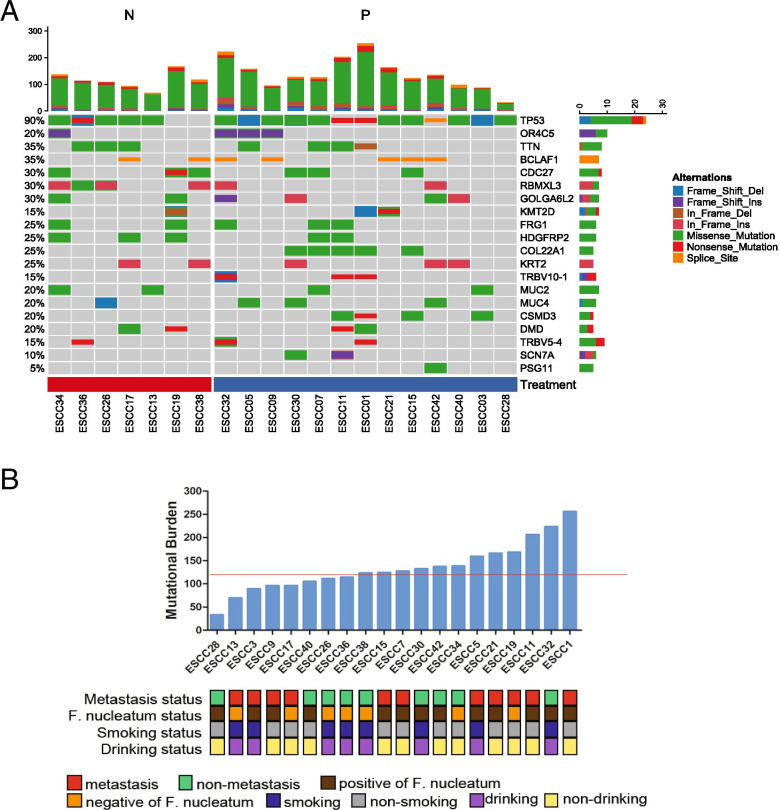
(**A**) Mutation frequencies and signatures, and significantly mutated genes in 20 ESCCs. The number of somatic mutations of each examined case (top) and the significantly mutated genes (SMGs) colored by the types of mutations and their mutational frequency (bottom). Columns, examined cases; rows, genes; N, *F. nucleatum*-negative group; P, *F. nucleatum*-positive group. (**B**) Combination of the mutational burden and the *F. nucleatum* content to predict metastasis in ESCC. The red horizontal line indicates the cutoff score for mutational burden. Clinical and pathological features that include the presence or absence of distant metastases in the surgical specimen are annotated for each patient. A sample was considered *F. nucleatum* positive if the qPCR Ct value was < 37 and the melt curve could be generated; otherwise, it was considered negative

MutSigCV analysis of 20 ESCC paired tissues revealed 20 significantly mutated genes (Fig. 7A). *TP53* mutations were present in all *F. nucleatum*-positive ESCCs. Most *TP53* mutations were in the hotspot exons 4–8. *TP53* is involved in the regulation of cell proliferation and apoptosis. Five of the 13 *F. nucleatum*-positive ESCCs harboring *TP53* mutations had concomitant mutations in *COL22A1*. The frequency of *F. nucleatum*-negative ESCC harboring *RBMXL3* and *HDGFRP2* mutations was 57.14 and 42.85%, respectively, which are different from the values for the *F. nucleatum*-positive group. Notably, we also identified a number of genes as occurring in only the *F. nucleatum*-positive group, including *COL22A1* (38.46%, 5/13), *TRBV10–1* (23.07%, 3/13), *CSMD3* (30.76%, 4/13), *SCN7A* (15.38%, 2/13) and *PSG11* (7.69%, 1/13) (Fig. 7A).

### Relationship between *F. nucleatum* and the mutational burden in ESCC

Of the 20 patients who provided samples for sequencing, 11 had distant metastasis within 6 months after the operation. There was no metastasis during 2 years of follow-up in the other 9 patients. Among these 11 patients, 72.8% (8/11) were *F. nucleatum* positive. In non-metastasis group, 55.6% (5/9) were *F. nucleatum* positive. A higher (mean ± SE) mutational burden was observed in the tumors of 11 patients with metastasis than those of patients without metastasis (141.5 ± 16.94 vs. 124 ± 16.31, *P* = 0.467), but there was no significant difference (Fig. 7B). The mutational burden per primary tumor was identified (median, 133.6; range, 33 to 256). The high-mutational-burden group was defined as patients with 120 or more mutations, and the low-mutational-burden group was defined as patients with less than 120 mutations. The results indicated that there was a certain correlation between the combination of the mutational burden and the *F. nucleatum* content in tumors and metastasis in ESCC, however, without significant difference.

## Discussion

The esophageal mucosa is among the sites colonized by the human microbiota, the complex microbial ecosystem that colonizes various body surfaces and is increasingly recognized to play roles in several physiological and pathological processes. To investigate the role of the microbiota of ESCC in different pathological stages, we performed 16S rRNA high-throughput sequencing in esophageal cancer tissues and normal tissues from 21 ESCC patients. The six major bacterial phyla (e.g., *Actinobacteria*, *Bacteroides*, *Firmicutes*, *Fusobacteria*, *Proteobacteria* and *Spirochaetes*) in these tissues are similar to the high-abundance bacterial types found in esophageal mucosa in previous studies [15]. *Proteobacteria*, *Firmicutes*, and *Actinobacteria* were the top 3 taxa that were significantly depleted in tumor tissues. In addition, specific bacteria are closely related to the progression of tumors in the pT stage and clinical stage, especially *Fusobacterium* and *Streptococcus,* which were also discovered to be enriched in the tumor group and may be among the important factors for the progression of ESCC. Previous studies on the diversity of bacterial flora between tumor and normal tissues have not been conclusive [16]. Bacterial diversity differs significantly according to cancer type. Some studies suggested that there were differences in the relative abundances of bacteria in oral squamous cell carcinoma (OSCC) patients; however, no statistically significant differences in phylogenies were detected for tumor and normal tissue sites except for the genus *Johnsonella* [17]. In contrast, colorectal tumor tissues harbor distinct microbial communities compared to nearby healthy tissue [18]. In this study, our analysis showed significant differences in the microbiota between tumor and normal tissues. The six major phyla of bacteria identified in tumor tissues are similar to those in normal or diseased esophageal mucosa, perhaps due to proximity [15]. However, they still show obvious clustering characteristics, which also implies that specific flora components exist in tumors and normal tissues, and identifying the difference between the two specific types of bacteria may be the key to understanding tumorigenesis.

Genus-level analysis showed that the abundances of *Fusobacterium* and *Streptococcus* increased significantly, while the abundances of *Butyrivibrio* and *Lactobacillus* decreased, in tumor tissues compared with those in normal tissues. *Butyrivibrio* species are considered nonpathogenic bacteria that can utilize cellulose, starch and other polysaccharides for chemical organic nutrition, and the main product of glucose fermentation by these species is butyric acid [19]. Butyric acid is an important short-chain fatty acid (SCFA) that can promote the growth of beneficial bacteria, such as *Lactobacillus*, in the body. *Lactobacillus* species are generally considered to be probiotics and can inhibit the growth of harmful bacteria [20]. The relative abundances of these two genera of bacteria decreased significantly, resulting in changes in the diversity of the microbiota in tissues and possibly increasing the number of pathogenic or harmful bacteria. Correspondingly, we found a significant increase in the abundances of *Fusobacterium* and *Streptococcus*, which are positively correlated with tumorigenesis and inflammation. Several studies have found that *Streptococcus gallolyticus* is related to colorectal cancer and can release toxins, regulate the tumor microenvironment or stimulate the immune response of immune cells [21, 22]. Moreover, *Streptococcus* abundance retained its association with unfavorable survival, suggesting that this may be an independent prognostic indicator for ESCC [23]. *Fusobacterium* is a genus that encompasses several species known to be opportunistic pathogens in humans; they are obligate anaerobes with known sites of infection in the oral cavity as well as in the gastrointestinal tract [24]. Relative *F. nucleatum* DNA levels in ESCC tissues compared with those in corresponding nontumor tissues were examined by qPCR, and FISH was used to analyze the distribution of *F. nucleatum* in ESCC tissue. Consistent with a previous study, the relative abundance of *F. nucleatum* was significantly higher in ESCC tissues than in adjacent nontumor tissues, which suggests that it is involved in the development of malignant tumors [12, 25]. One possibility is that these bacteria may play a role in the etiology of ESCC; another possibility is that these species are enriched as the tumor has formed a niche that favors these bacteria.

Furthermore, we explored the relationship between specific microbiota composition and the pathological development of ESCC. In regard to pT stage, previous studies have shown that the abundance of only *Streptococcus* in pT3–4 was significantly higher than that in pT1–2 of ESCC, while the other genera showed no significant change [23]. However, in our results, *Fusobacteria* and *Bacteroidetes* were found in pT3–4 at the phylum level, and the relative abundance of *F. nucleatum,* an important representative strain of *Fusobacterium*, in pT3–4 was also significantly higher than that in pT1–2. As a strictly anaerobic gram-negative bacterium, *F. nucleatum* is mainly distributed in human and animal intestines and in the oral mucosa. *F. nucleatum* is the most abundant species in the oral cavity and has come to the forefront of scientific interest because of an increasing number of associations with extraoral diseases [6]. In many studies, it has been shown that *F. nucleatum* is closely related to the occurrence of tumors, especially colon cancer [5, 12]. In addition, it is positively related to the poor prognosis of esophageal cancer [12]. Recently, research has found that a higher *F. nucleatum* burden correlates with a poor response to neoadjuvant chemotherapy in ESCC [13]. Meanwhile, fecal *F. nucleatum* as a predictor for metachronous colorectal adenoma after endoscopic polypectomy [26]. According to previous studies on *F. nucleatum*, combined with the different relative abundances of *F. nucleatum* in different pT and clinical stages of ESCC in this study, *F. nucleatum* might be an important factor in the tumor progression of ESCC. This also provides a new way for us to understand the causes of the occurrence and development of ECSS.

Gene mutation is an important factor that affects signal pathway regulation and participates in tumor pathogenesis. Accumulated gene mutations accelerate genome instability, which eventually leads to uncontrollable growth of the tumor. However, it is not clear whether *F. nucleatum* affects the progression of ESCC by inducing gene mutations. It is hypothesized that bacterial species such as *Escherichia coli*, *F. nucleatum*, and enterotoxigenic *Bacteroides fragilis* have a role in colorectal carcinogenesis. In addition, bacteria produce toxins that inhibit the immune response and cause DNA damage [27]. However, *F. nucleatum* encodes no known toxins and very few canonical ‘virulence factors’ [6]. However, epidemiological associations suggest that *F. nucleatum* can promote genome instability and mutation. A previous study showed that *TP53*, *KRAS*, and *BRAF* mutations were additionally associated with *F. nucleatum*-positive colorectal cancers (CRCs) [28]. We analyzed 13 samples of *F. nucleatum*-positive ESCC by WES and found that the function of the mutant gene is mainly concentrated in the pathway of positive regulation of apoptosis and the epidermal growth factor-like protein domain. Cancer cells have developed mechanisms by which apoptosis is evaded through the mutation of essential genes involved in the regulation of the process. Epidermal growth factor (EGF) and its receptor play an important role in signaling pathways and in regulating cell proliferation, migration, differentiation and apoptosis [29]. We found a common mutant gene, *TP53,* in 13 *F. nucleatum*-positive ESCC tumor tissues, and we found genes that were present only in *F. nucleatum*-positive tumor tissues, such as *COL22A1, TRBV10–1, CSMD3, SCN7A* and *PSG11*. This finding is different from previous reports showing that *KRAS* and *BRAF* frequently occur in CRCs [28]. We infer that this may be caused by the small sample size and different cancer types. Nevertheless, our results support the relationship between *F. nucleatum* and tumor genetics. We advocate the need for large-scale research and further systems analysis to obtain conclusive evidence.

Tumor mutation burden (TMB), a quantitative measure of the total number of coding mutations in the tumor genome, is emerging as a potential biomarker. Both exogenous DNA damage caused by DNA-damaging agents and endogenous DNA damage caused by increased production of reactive oxygen species could produce gene mutations. Specific mutations in tumor cells may lead to peptide epitopes or tumor-specific antigens for T-cells, and these antigens could serve as immunotherapeutic targets. The number of somatic mutations varies widely both between and within cancer types. Previous clinical trials in multiple tumors have also shown that there is a positive correlation between TMB and the efficacy of PD-1/PD-L1 inhibitors and a trend toward longer PFS [30, 31]. Li et al found that a high TMB was significantly associated with a worse prognosis and could promote tumor metastasis and development [32]. In this study, we found that *F. nucleatum* was positively correlated with the degree of malignancy of ESCC. Meanwhile, the present results suggest that the combined analysis of *F. nucleatum* positive and high TMB in tumor tissues is expected to predict the prognosis and metastasis of ESCC.

## Conclusion

In conclusion, we found that the abundance of *F. nucleatum* in ESCC tissue is closely related to the development of ESCC tumor tissues in the pT and clinical stages, suggesting that it may play an important role in the progression of ESCC. *F. nucleatum* might participate in the mechanisms by which epidermal growth factor (EGF) is induced to interfere with the cell apoptosis-mediated regulation of related genes in this process. Importantly, preliminary data results provide us with a new insight that the abundance of *F. nucleatum* and the TMB might be used in combination as a potential method to predict the potential of metastasis in ESCC.

## Methods

### Sample collection and ethical statement

We retrospectively collected 152 surgical specimens (including 82 tumor and paired non-tumor tissue samples from 41 patients, and 70 individual ESCC tumor tissue samples) from 111 patients at Henan Cancer Hospital affiliated to Zhengzhou University from 2016 to 2017. These patients were diagnosed with primary ESCC, which were confirmed by postoperative pathology. Exclusion criteria included patients who had received preoperative radiotherapy, chemotherapy or had a previous history of ESCC or inflammatory bowel disease. Retrospective clinic-pathological data were collected, including gender, age, alcohol consumption, smoking, tumor site, histological grade, clinical pT stage, lymph node metastasis stage, and TNM stage, according to the 7th edition of the Union for International Cancer Control (UICC)/TNM. The characteristics of this study patients are shown in Table 1. The study was approved by the Institutional Ethics Committee of Henan Cancer Hospital affiliated with Zhengzhou University (Approval number 2017407) and all surgical tissue were collected after obtaining informed and written consents from the patients.

### DNA extraction and 16S ribosomal RNA sequencing

DNA was extracted from each specimen in whole tissue sections of FFPE tissue blocks using QIAamp DNA FFPE Tissue Kit (QIAGEN, Germany) according to the manufacturer’s instructions. We quantified extracted DNA using High Sensitivity Qubit (Thermofisher, Carlsbad, CA) and amplified 30 ng of DNA using specific barcoded primers around the V3-V4 16S rRNA (336F: GTACTCCTACGGGAGGCAGCA; 806R: GTGGACTACHVGGGTWTCTAAT). We then sequenced the amplified samples using the 16S rRNA high-throughput next generation Illumina MiSeq (250 PE, San Diego, CA) platform in Allwegene company (Beijing, China).

### Analysis of 16S rRNA amplicon sequence data

Quantitative insights into microbial Ecology (QIIME) were used to analyze the sequencing data. We selected operational taxonomic units (OTUs) at 97% similarity, and trimmed to span only the 16S rRNA region flanked by our sequencing primers, which cross-referenced with Greengenes and SilVA128 databases. In addition, magic-BLAST was used to eliminate human genome noise in host DNA contamination. Trimmomatic (V0.32) was required to trim FASTQ data, quality check and Cutadapt removed adapter sequences and primers. Dada2 was used for relative abundance, alpha−/beta diversity and sample cluttering heatmap analyses. The QIIME script “beta_diversity_through_plost.py” was performed for Bray-Curtis principal coordinate analysis.

### Quantitative polymerase chain reaction (qPCR) assays

We determined the amount of *F. nucleatum* DNA by real-time qPCR assay. Custom primer sets for the *nusG* gene of *F. nucleatum* and for the reference human gene, *SLCO2A1* were used as previously described [12, 13]: *nusG* forward primer, 5′-TGGTGTCATTCTTCCAAAAATATCA-3′; *nusG* reverse primer, 5′-AGATCAAGAAGGACAAGTTGCTGAA-3′; *SLCO2A1* forward primer, 5′-ATCCCCAAAGCACCTGGTTT-3′; *SLCO2A1* reverse primer, 5′-AGAGGCCAAGATAGTCCTGGTAA-3′; Each reaction contained 40 ng of genomic DNA and was assayed in 10 μL reactions containing 1 × Power SYBR Green PCR Master Mix (Applied Biosystems, Carlsbad, CA, USA), 0.4 μM each primer and was placed in a 96-well optical PCR plate [33]. Amplification and detection of DNA was performed with the StepOnePlus real-time PCR Systems (Applied Biosystems) using the following reaction conditions: 10 min at 95 °C, 45 cycles of 15 s at 95 °C and 1 min at 60 °C. The detectable amount of tissue *F. nucleatum* DNA was calculated as a relative unitless value normalised with *SLCO2A1* using the 2^−ΔCt^ method (where ΔCt= ‘the mean Ct value of *F. nucleatum*’- ‘the mean Ct value of *SLCO2A1*’) [5]. The reliability of qPCR results was evaluated by the dissolution curve, and the Ct value (inflection point of the expanded dynamic curve) was taken. In our study *F. nucleatum*-positive was defined as the sample with a Ct less than 37, while the samples with a Ct value greater than 37 were considered as negative.

### FISH analysis

The probes used in this study were as follows: an Oregon-Green 488-conjugated “universal bacterial” probe (EUB338, pB-00159, green) binding 16S rRNA gene at bacterial conserved regions and a Cy3conjugated Fusobacterium probe (FUSO, pB-2634, red) binding 16S rRNA gene at *Fusobacterium* specific regions. The sequences of the probes were referred to probeBase (http://www.microbialecology.net/ probebase) [34, 35]. The probe cocktail, which contained 100 to 120 ng of each probe (Sangon, Shanghai, China), was resuspended in a hybridization buffer. FISH analysis was performed as previously described [36]. Any sample was evaluated by two pathologists blindly who chosen five random × 40 fields in each status.

### Whole-exome sequencing (WES) and data analysis

Tumor and normal genomic DNA from formalin-fixed, paraffin-embedded tissue samples was extracted (Qiagen, Hilden, Germany). The nucleic acids of 21 pairs samples were subjected to quantitative and qualitative evaluation, using the NanoDrop instrument, and the Agilent Bioanalyzer (Agilent Technologies, Santa Clara USA), respectively. All the final DNA library were subsequently sequencing on the MGISEQ-2000 high-throughput platform, according to the manufacturer’s instructions. The DNBs were loaded into the patterned nanoarrays and pair-end read of 100 bp were read through on the MGISEQ-2000 platform for the following data analysis study. Sequencing data was aligned to the human reference genome (hg19) after removal of low-quality reads, using the BWA version 0.5.9 (Broad Institute) with default parameters. Sequencing data from paired tumor-normal samples were used to identify somatic mutations. After annotation, the variants were cross referenced with those in the 1000 Genomes Project, GAD, dbSNP, and the ExAC. Variants with an allele prevalence > 1% in the 1000 genomes project, 1000 genomes project EAS, ExAC, ExAC EAS were excluded. MuTect (version 1.1.4) and NChot were performed for identifying single-nucleotide variants. And small insertions and deletions were determined by GATK. Copy number variations were detected through the CONTRA tool (2.0.8). At least five supporting reads were required for true fusion.

### Statistical analysis

Statistical analysis and drawing were performed using the GraphPad Prism 8 (GraphPad, San Diego, California, USA). Significant among groups was analyzed by One-way ANOVA. Unpaired t-test and paired t-test are used for comparing depending on whether the samples are paired. A two-sided *P* value < 0.05 was considered statistically significant. The biological functions of the related genes were explored by GO and pathway enrichment analysis in DAVID Bioinformatics Resources 6.8. The tumor group and the normal group were clustered by principal coordinate analysis, tree clustering analyses, cluster dendrogram with AU/BP values.

## Supplementary Information

**Additional file 1:.**

## Data Availability

The 16S rRNA sequencing datasets generated and/or analyzed during the current study are available from Sequence Read Archive (SRA) repository (accession number: PRJNA766558). Human Whole-Exome Sequencing (WES) datasets are available from the corresponding author on reasonable request.

## Abbreviations

ESCC: Esophageal squamous cell carcinoma
16S rRNA: 16S ribosomal RNA
OTUs: Operational taxonomic units
QIIME: Quantitative insights into microbial ecology
N: Normal group
T: Tumor group
G: Tumor grades
pT: Tumor invasive depth
GO: Gene Ontology
WES: Whole exome sequencing
TMB: Tumor mutation burden
