# Supplementary material for: Fusobacterium nucleatum predicts a high risk of metastasis for esophageal squamous cell carcinoma
Source: BMC Microbiol. 2021 Oct 30;21:301. doi: 10.1186/s12866-021-02352-6 (PMC8556942; doi:10.1186/s12866-021-02352-6)

**Supplementary Material**

***Fusobacterium nucleatum* predicts a risk for esophageal squamous cell carcinoma**

Running title: *F. nucleatum* predicts a risk of metastasis for ESCC

^#^Zhen Li^1,2^; ^#^Chao Shi^3,4^; Jiawen Zheng^3,4^; Yongjun Guo^3,4^; Taibing Fan^1^; Huan Zhao^5^; Dongdong Jian^1^; Xiaolei Cheng^1^; Hao Tang^1*^; Jie Ma^3,4*^

Additional Supporting Information may be found in the online version of this article at the publisher’s website:

**Table S1**. Signature microbiota profiles in the comparison of norma and tumor groups at phylum, class and genus levels

| **Kingdom** | **Phylum** | **Class** | **Order** | **Family** | **Genus** | **P-value** |
| --- | --- | --- | --- | --- | --- | --- |
| Bacteria | *Proteobacteria* | *Gammaproteobacteria* | *Enterobacteriales* | *Enterobacteriaceae* | *Klebsiella* | 2.10E-11 |
| Bacteria | *Firmicutes* | *Clostridia* | *Clostridiales* | *Lachnospiraceae* | *Lachnospiraceae_NK4A136_group* | 2.91E-09 |
| Bacteria | *Firmicutes* | *Clostridia* | *Clostridiales* | *Lachnospiraceae* | NA | 1.29E-08 |
| Bacteria | *Firmicutes* | *Clostridia* | *Clostridiales* | *Lachnospiraceae* | NA | 2.63E-08 |
| Bacteria | *Proteobacteria* | *Gammaproteobacteria* | *Enterobacteriales* | *Enterobacteriaceae* | *Klebsiella* | 4.66E-08 |
| Bacteria | *Proteobacteria* | *Alphaproteobacteria* | *Sphingomonadales* | *Sphingomonadaceae* | *Sphingomonas* | 1.02E-07 |
| Bacteria | *Proteobacteria* | *Alphaproteobacteria* | *Rhizobiales* | *Beijerinckiaceae* | *Methylobacterium* | 5.13E-06 |
| Bacteria | *Actinobacteria* | *Actinobacteria* | *Micrococcales* | NA | NA | 7.65E-06 |
| Bacteria | *Proteobacteria* | *Gammaproteobacteria* | *Betaproteobacteriales* | *Burkholderiaceae* | *Parasutterella* | 2.32E-05 |
| Bacteria | *Actinobacteria* | *Actinobacteria* | *Pseudonocardiales* | *Pseudonocardiaceae* | *Pseudonocardia* | 3.04E-05 |
| Bacteria | *Firmicutes* | *Clostridia* | *Clostridiales* | *Lachnospiraceae* | *Lachnospiraceae_NK4A136_group* | 8.79E-05 |
| Bacteria | *Actinobacteria* | *Actinobacteria* | *Corynebacteriales* | *Corynebacteriaceae* | *Lawsonella* | 0.000197 |
| Bacteria | *Actinobacteria* | *Actinobacteria* | *Propionibacteriales* | *Propionibacteriaceae* | *Cutibacterium* | 0.000211 |
| Bacteria | *Firmicutes* | *Bacilli* | NA | NA | NA | 0.000287 |
| Bacteria | *Firmicutes* | *Bacilli* | *Bacillales* | *Staphylococcaceae* | *Staphylococcus* | 0.000318 |
| Bacteria | *Proteobacteria* | *Gammaproteobacteria* | *Enterobacteriales* | *Enterobacteriaceae* | *Escherichia/Shigella* | 0.000361 |
| Bacteria | *Actinobacteria* | *Actinobacteria* | *Corynebacteriales* | *Corynebacteriaceae* | *Corynebacterium* | 0.000368 |
| Bacteria | *Proteobacteria* | *Gammaproteobacteria* | *Pseudomonadales* | *Moraxellaceae* | *Enhydrobacter* | 0.000441 |
| Bacteria | *Firmicutes* | *Bacilli* | *Lactobacillales* | *Lactobacillaceae* | *Lactobacillus* | 0.00052 |
| Bacteria | *Actinobacteria* | *Actinobacteria* | *Micrococcales* | *Brevibacteriaceae* | *Brevibacterium* | 0.000732 |
| Bacteria | *Actinobacteria* | *Actinobacteria* | *Corynebacteriales* | *Corynebacteriaceae* | *Corynebacterium_1* | 0.000943 |
| Bacteria | *Proteobacteria* | *Alphaproteobacteria* | *Rhizobiales* | *Xanthobacteraceae* | NA | 0.001109 |
| Bacteria | *Proteobacteria* | *Gammaproteobacteria* | *Betaproteobacteriales* | *Burkholderiaceae* | *Massilia* | 0.001151 |
| Bacteria | *Proteobacteria* | *Gammaproteobacteria* | *Betaproteobacteriales* | *Burkholderiaceae* | *Lautropia* | 0.001241 |
| Bacteria | *Firmicutes* | *Bacilli* | *Lactobacillales* | *Streptococcaceae* | *Streptococcus* | 0.001334 |
| Bacteria | *Actinobacteria* | *Acidimicrobiia* | *Microtrichales* | *Ilumatobacteraceae* | NA | 0.001365 |
| Bacteria | *Actinobacteria* | *Actinobacteria* | *Corynebacteriales* | *Corynebacteriaceae* | *Lawsonella* | 0.001593 |
| Bacteria | *Proteobacteria* | *Gammaproteobacteria* | *Betaproteobacteriales* | *Burkholderiaceae* | *Limnobacter* | 0.001597 |
| Bacteria | *Firmicutes* | *Clostridia* | *Clostridiales* | *Lachnospiraceae* | NA | 0.001673 |
| Bacteria | *Proteobacteria* | *Alphaproteobacteria* | *Caulobacterales* | *Caulobacteraceae* | *Brevundimonas* | 0.001819 |
| Bacteria | *Proteobacteria* | *Gammaproteobacteria* | *Enterobacteriales* | *Enterobacteriaceae* | *Pluralibacter* | 0.001876 |
| Bacteria | *Proteobacteria* | *Gammaproteobacteria* | *Pseudomonadales* | *Pseudomonadaceae* | *Pseudomonas* | 0.001911 |
| Bacteria | *Firmicutes* | *Bacilli* | *Lactobacillales* | *Carnobacteriaceae* | *Atopostipes* | 0.002462 |
| Bacteria | *Proteobacteria* | *Gammaproteobacteria* | *Pseudomonadales* | *Moraxellaceae* | *Acinetobacter* | 0.002498 |
| Bacteria | *Firmicutes* | *Clostridia* | *Clostridiales* | *Lachnospiraceae* | *Lachnospiraceae_NK4A136_group* | 0.002846 |
| Bacteria | *Proteobacteria* | *Gammaproteobacteria* | *Betaproteobacteriales* | *Neisseriaceae* | *Neisseria* | 0.002894 |
| Bacteria | *Bacteroidetes* | *Bacteroidia* | *Flavobacteriales* | *Flavobacteriaceae* | *Flavobacterium* | 0.002901 |
| Bacteria | *Proteobacteria* | *Gammaproteobacteria* | *Pseudomonadales* | *Moraxellaceae* | *Acinetobacter* | 0.003132 |
| Bacteria | *Proteobacteria* | *Gammaproteobacteria* | *Pseudomonadales* | *Pseudomonadaceae* | *Pseudomonas* | 0.003142 |
| Bacteria | *Firmicutes* | *Clostridia* | *Clostridiales* | *Peptostreptococcaceae* | *Peptostreptococcus* | 0.003537 |
| Bacteria | *Firmicutes* | *Clostridia* | *Clostridiales* | *Lachnospiraceae* | *Lachnospiraceae_NK4A136_group* | 0.003708 |
| Bacteria | *Firmicutes* | *Bacilli* | *Lactobacillales* | *Carnobacteriaceae* | *Atopostipes* | 0.003941 |
| Bacteria | *Firmicutes* | *Bacilli* | *Bacillales* | *Staphylococcaceae* | *Staphylococcus* | 0.00411 |
| Bacteria | *Firmicutes* | *Bacilli* | *Bacillales* | *Staphylococcaceae* | *Staphylococcus* | 0.004828 |
| Bacteria | *Proteobacteria* | *Gammaproteobacteria* | *Enterobacteriales* | *Enterobacteriaceae* | *Klebsiella* | 0.004951 |
| Bacteria | *Fusobacteria* | *Fusobacteriia* | *Fusobacteriales* | *Fusobacteriaceae* | *Fusobacterium* | 0.005224 |
| Bacteria | *Firmicutes* | *Clostridia* | *Clostridiales* | *Lachnospiraceae* | *Lachnoclostridium* | 0.005595 |
| Bacteria | *Proteobacteria* | *Gammaproteobacteria* | *Betaproteobacteriales* | *Burkholderiaceae* | *Delftia* | 0.005693 |
| Bacteria | *Actinobacteria* | *Actinobacteria* | *Actinomycetales* | *Actinomycetaceae* | *Actinomyces* | 0.006516 |
| Bacteria | *Firmicutes* | *Bacilli* | *Lactobacillales* | *Lactobacillaceae* | *Lactobacillus* | 0.006664 |
| Bacteria | *Firmicutes* | *Clostridia* | *Clostridiales* | *Lachnospiraceae* | *Lachnospiraceae_NK4A136_group* | 0.007205 |
| Bacteria | *Actinobacteria* | *Actinobacteria* | *Corynebacteriales* | *Corynebacteriaceae* | *Corynebacterium_1* | 0.007597 |
| Bacteria | *Firmicutes* | *Bacilli* | *Lactobacillales* | *Carnobacteriaceae* | *Dolosigranulum* | 0.007696 |
| Bacteria | *Firmicutes* | *Clostridia* | *Clostridiales* | *Lachnospiraceae* | *Butyrivibrio* | 0.007814 |
| Bacteria | *Firmicutes* | *Bacilli* | *Lactobacillales* | *Aerococcaceae* | *Aerococcus* | 0.008266 |

**Table S2**. Differential analyses of pT stage patients

|  |  |  |  |  |  | **Comparison:**  **pT3 vs pT1** |  |  | **Comparison:**  **pT3 vs pT2** |  |  |
| --- | --- | --- | --- | --- | --- | --- | --- | --- | --- | --- | --- |
| **Kingdom** | **Phylum** | **Class** | **Order** | **Family** | **Genus** | **log2FoldChange** | **Pvalue** | **Padj** | **log2FoldChange** | **Pvalue** | **Padj** |
| Bacteria | *Firmicutes* | *Bacilli* | *Lactobacillales* | *Lactobacillaceae* | *Lactobacillus* | -24.81 | 1.25E-16 | 6.12E-15 | -22.38 | 2.89E-16 | 1.83E-14 |
| Bacteria | *Proteobacteria* | *Alphaproteobacteria* | *Acetobacterales* | *Acetobacteraceae* | *Acetobacter* | -25.12 | 5.77E-17 | 5.64E-15 | -22.29 | 4.21E-16 | 1.83E-14 |
| Bacteria | *Bacteroidetes* | *Bacteroidia* | *Cytophagales* | *Cyclobacteriaceae* | *Algoriphagus* | -24.64 | 5.81E-10 | 1.06E-08 | -21.77 | 1.73E-09 | 2.21E-08 |
| Bacteria | *Proteobacteria* | *Gammaproteobacteria* | *Oceanospirillales* | *Halomonadaceae* | *Carnimonas* | -23.82 | 2.43E-13 | 5.92E-12 | -20.81 | 2.50E-12 | 5.36E-11 |
| Bacteria | *Proteobacteria* | *Gammaproteobacteria* | *Betaproteobacteriales* | *Burkholderiaceae* | *Hydrogenophaga* | -23.69 | 1.89E-14 | 6.91E-13 | -21.37 | 3.97E-14 | 1.08E-12 |
| Bacteria | *Proteobacteria* | *Gammaproteobacteria* | *Betaproteobacteriales* | *Burkholderiaceae* | *Methylibium* | -24.67 | 2.34E-13 | 5.92E-12 | -21.07 | 7.02E-12 | 1.09E-10 |
| Bacteria | *Proteobacteria* | *Gammaproteobacteria* | *Betaproteobacteriales* | *Methylophilaceae* | *Methylophilus* | -24.77 | 8.88E-17 | 6.12E-15 | -20.70 | 3.19E-14 | 9.95E-13 |
| Bacteria | *Proteobacteria* | *Gammaproteobacteria* | *Betaproteobacteriales* | *Methylophilaceae* | *Methylotenera* | -24.61 | 1.64E-13 | 4.97E-12 | -20.06 | 5.02E-11 | 6.84E-10 |
| Bacteria | *Actinobacteria* | *Actinobacteria* | *Corynebacteriales* | *Mycobacteriaceae* | *Mycobacterium* | -25.78 | 9.13E-11 | 1.91E-09 | -24.10 | 2.57E-11 | 3.74E-10 |
| Bacteria | *Actinobacteria* | *Actinobacteria* | *PeM15* | *NA* | *NA* | -29.78 | 1.57E-14 | 6.57E-13 | -28.98 | 2.00E-16 | 1.83E-14 |
| Bacteria | *Bacteroidetes* | *Bacteroidia* | *Chitinophagales* | *Saprospiraceae* | *NA* | -24.22 | 6.33E-13 | 1.43E-11 | -21.07 | 6.88E-12 | 1.09E-10 |
| Bacteria | *Proteobacteria* | *Gammaproteobacteria* | *Betaproteobacteriales* | *Burkholderiaceae* | *Noviherbaspirillum* | -27.96 | 1.23E-18 | 2.10E-16 | -23.56 | 3.81E-16 | 1.83E-14 |
| Bacteria | *Proteobacteria* | *Alphaproteobacteria* | *Sphingomonadales* | *Sphingomonadaceae* | *Novosphingobium* | -26.56 | 1.20E-16 | 6.12E-15 | -23.27 | 1.82E-15 | 6.61E-14 |
| Bacteria | *Bacteroidetes* | *Bacteroidia* | *Bacteroidales* | *Prevotellaceae* | *Prevotella_7* | 9.14 | 1.41E-03 | 1.88E-02 | 8.85 | 6.06E-04 | 6.29E-03 |
| Bacteria | *Proteobacteria* | *Gammaproteobacteria* | *Betaproteobacteriales* | *Burkholderiaceae* | *Ramlibacter* | -26.34 | 1.43E-18 | 2.10E-16 | -22.53 | 1.73E-16 | 1.83E-14 |
|  |  |  |  |  |  | **Comparison:**  **pT4 vs pT1** |  |  | **Comparison:**  **pT4 vs pT2** |  |  |
| **Kingdom** | **Phylum** | **Class** | **Order** | **Family** | **Genus** | **log2FoldChange** | **Pvalue** | **Padj** | **log2FoldChange** | **Pvalue** | **Padj** |
| Bacteria | *Proteobacteria* | *Gammaproteobacteria* | *Pasteurellales* | *Pasteurellaceae* | *Actinobacillus* | -20.17 | 9.67E-05 | 0.0087 | -15.85 | 0.0017 | 0.037 |
| Bacteria | *Fusobacteria* | *Fusobacteriia* | *Fusobacteriales* | *Fusobacteriaceae* | *Fusobacterium* | -21.75 | 1.69E-05 | 0.0027 | -16.27 | 0.00095 | 0.031 |
| Bacteria | *Firmicutes* | *Bacilli* | *Bacillales* | *Family_XI* | *Gemella* | -21.63 | 1.27E-07 | 4.00E-05 | -18.87 | 2.29E-06 | 0.00035 |
| Bacteria | *Proteobacteria* | *Alphaproteobacteria* | *Sphingomonadales* | *Sphingomonadaceae* | *Novosphingobium* | -20.64 | 0.00018 | 0.011 | -17.35 | 0.0012 | 0.031 |

**Table S3**. **Microbiota associated with clinical characters in ESCC**

| Clinical Characters | Age  (<60 vs≥60) | Gender  (Male vs Female) | Smoking  (Yes or No) | Drinking  (Yes or No) | Lymph node metastasis  (Yes or No) | Tumor location  (Top vs Middle down) | pT stage  (pT1-2 vs pT3-4) | Clinical tumor stage  (I-II vs III) | G stage  (G1-2 vs G3) |
| --- | --- | --- | --- | --- | --- | --- | --- | --- | --- |
| Mean Value |  |  |  |  |  |  |  |  |  |
| Chao1 | 998.12 vs 1019.43 | 978.85 vs 1042.29 | 996.88 vs 1019.62 | 1028.43 vs 968.48 | 679.54 vs 1052.507 | 1297.66 vs 952.97 | 1094.03 vs 843.77 | 1011.25 vs 984.73 | 950.03 vs 988.71 |
| Shannon index | 5.54 vs 5.69 | 5.62 vs 5.68 | 5.53 vs 5.76 | 5.72 vs 5.54 | 4.96 vs 5.75 | 6.07 vs 5.55 | **5.86 vs 5.22*** | 5.67 vs 5.64 | 5.33 vs 5.54 |
| PD_whole_tree | 488.09 vs 474.33 | 509.35 vs 421.33 | 509.44 vs 441.94 | 521.15 vs 420.91 | 375.97 vs 495.69 | 602.67 vs 451.27 | 493.81 vs 450.37 | 480.52 vs 464.56 | 422.13 vs 491.23 |
| Observed species | 729.48 vs 734.63 | 732.41 vs 726.89 | 740.55 vs 724.81 | 763.69 vs 695.29 | 509.5 vs 769.91 | 921.18 vs 693.51 | 790.09 vs 626.27 | 741.26 vs 720.91 | 681.74 vs 741.78 |

**P-*value <0.05.

**Figure S1. The rarefaction curve and the relative abundance of bacteria at class levels in both N and T groups.**


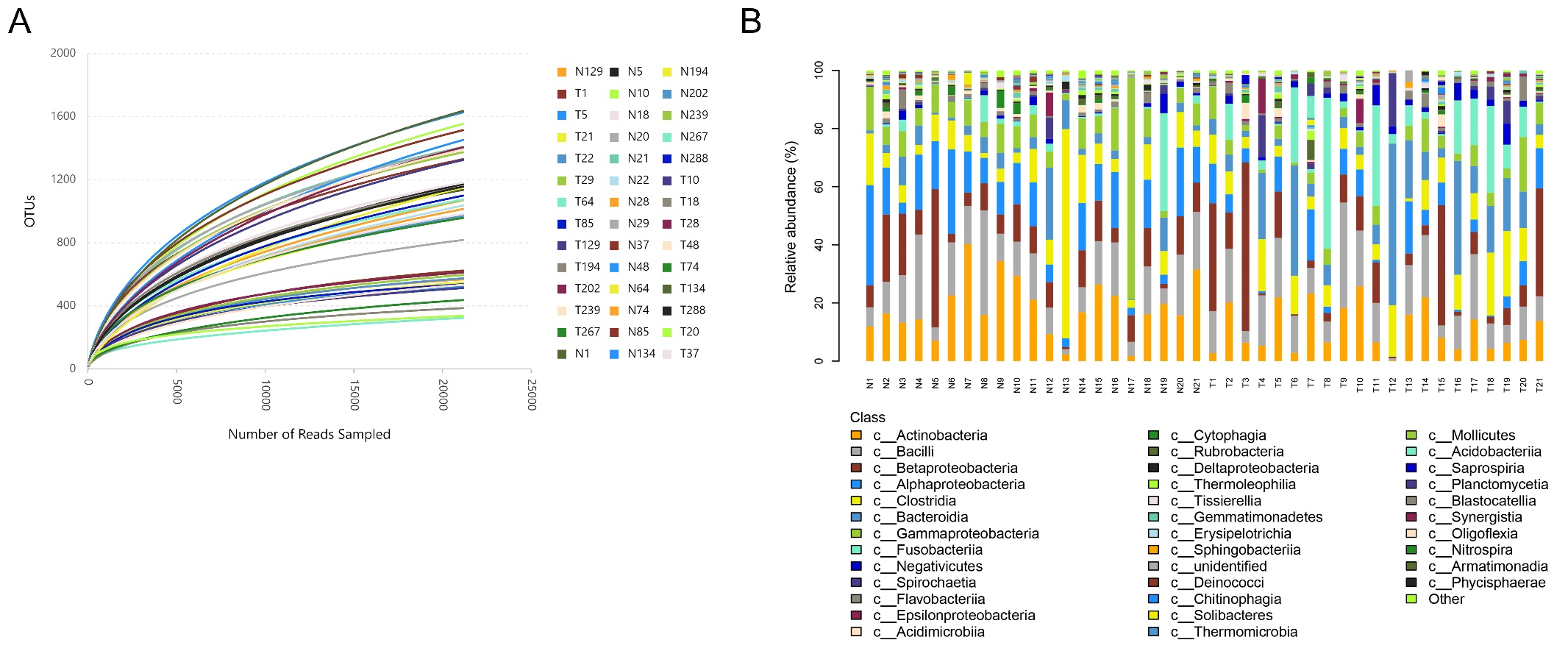


**Figure S2.** *Fusobacterium* is anaerobic, gram-negative bacteria and normally treated as pathogen, which discovered as increasing relative abundance in tumor groups (*P*=0.0052).


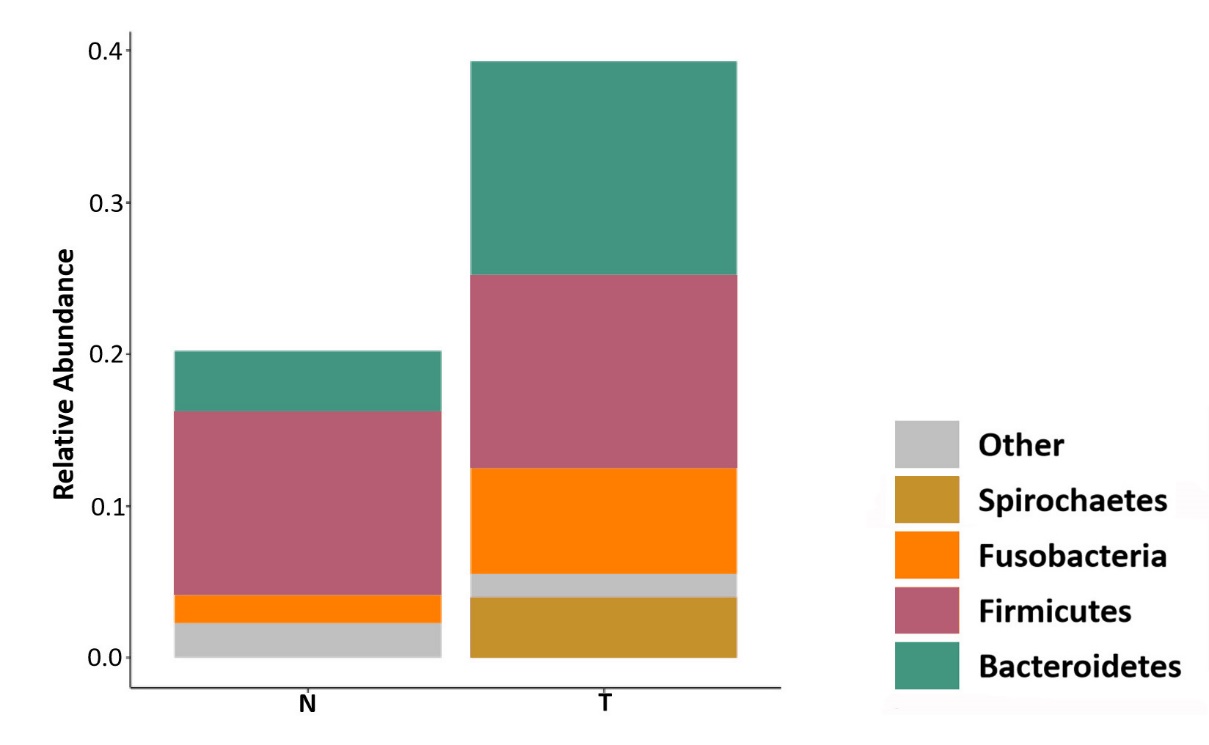


**Figure S3.** The results of heatmap (A) showed that the relative abundance of *Fusobacterium* (*P=*0.039) and *Prevotella* (*P=*0.0379) were corrected with clinical stage in ESCC, where were higher in tumor than in corresponding normal tissues (B), without IA stage.


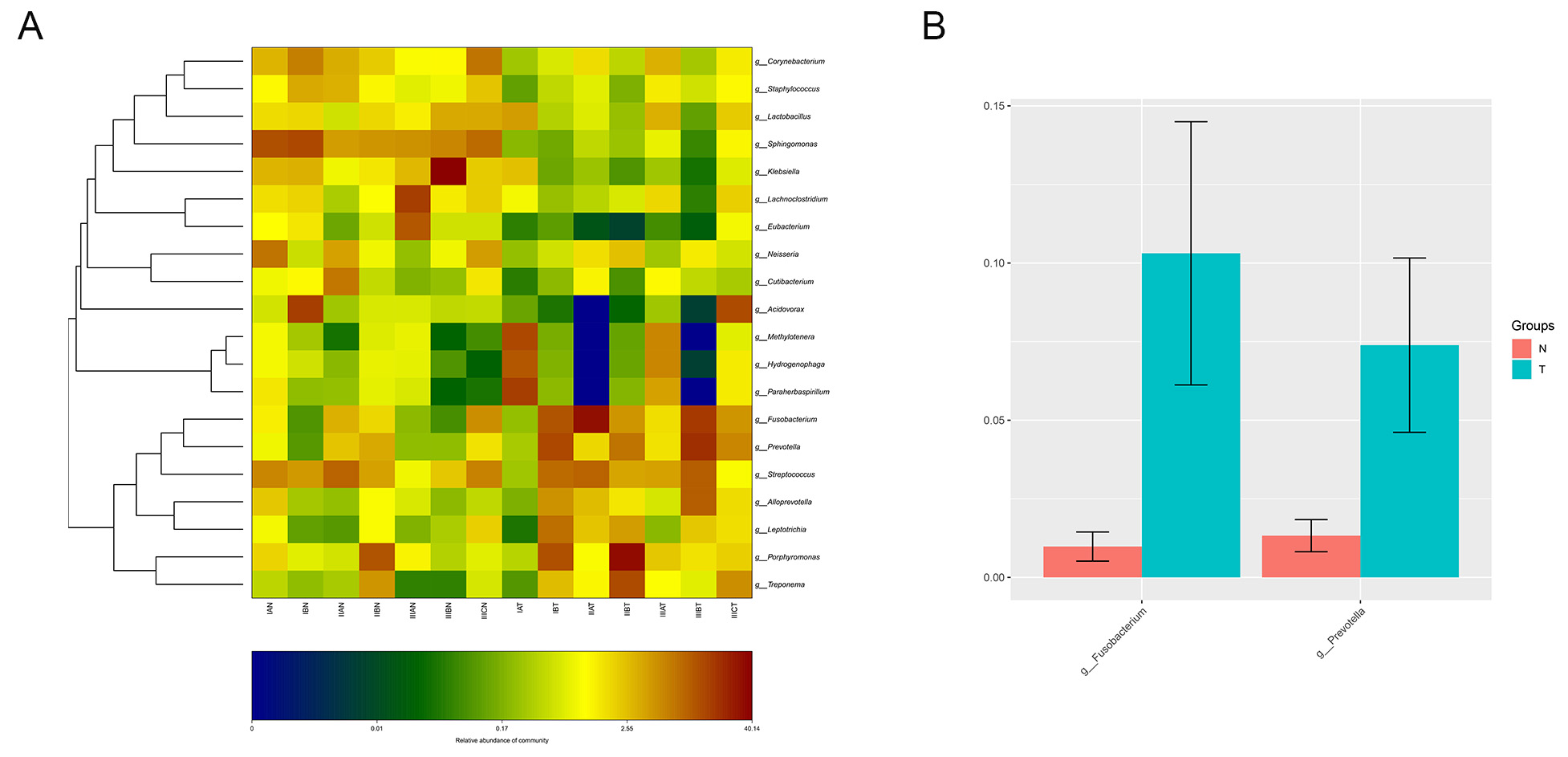


**Figure S4. *Fusobacteria* and *Bacteroidetes* increased significantly in pT3-4.** Group A consisted of pT1 and pT2, Group B consisted of pT3 and pT4. Gram-negative bacteria(A), gram-positive bacteria(B) and anaerobic bacteria(C) in the flora were taken as the object of analysis, respectively.


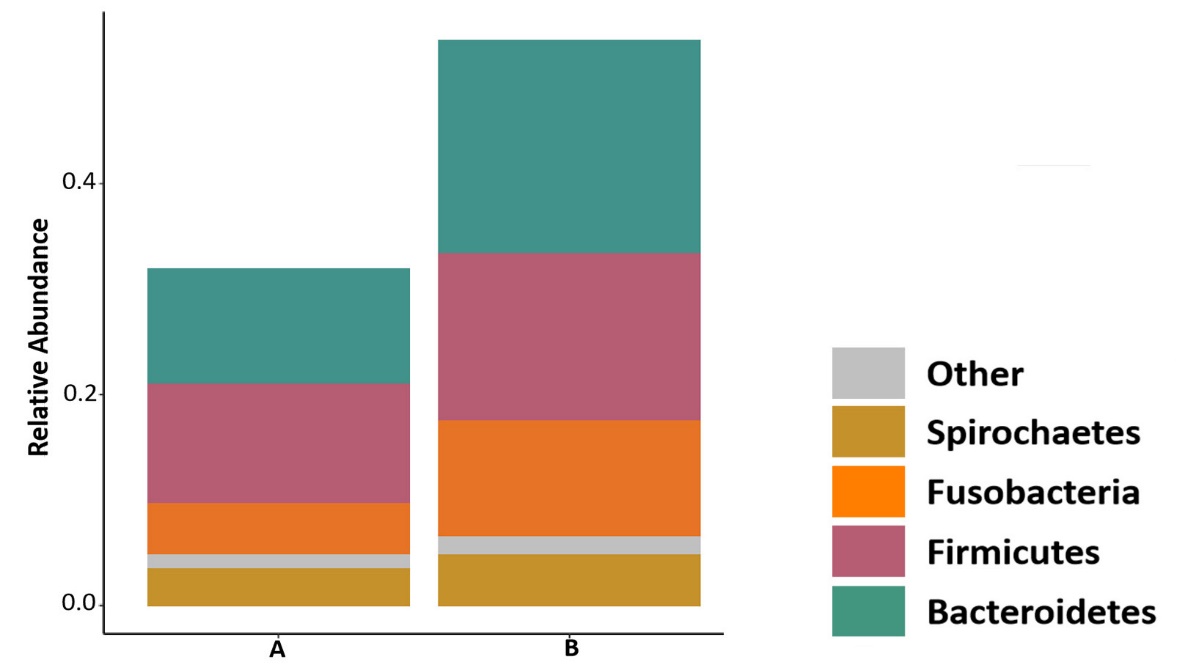


**Figure S5.** The relative *F. nucleatum* DNA levels in ESCC tumor tissues had no significant difference among patient's age, gender, smoking history, drinking history, lymph node metastasis and G stage.


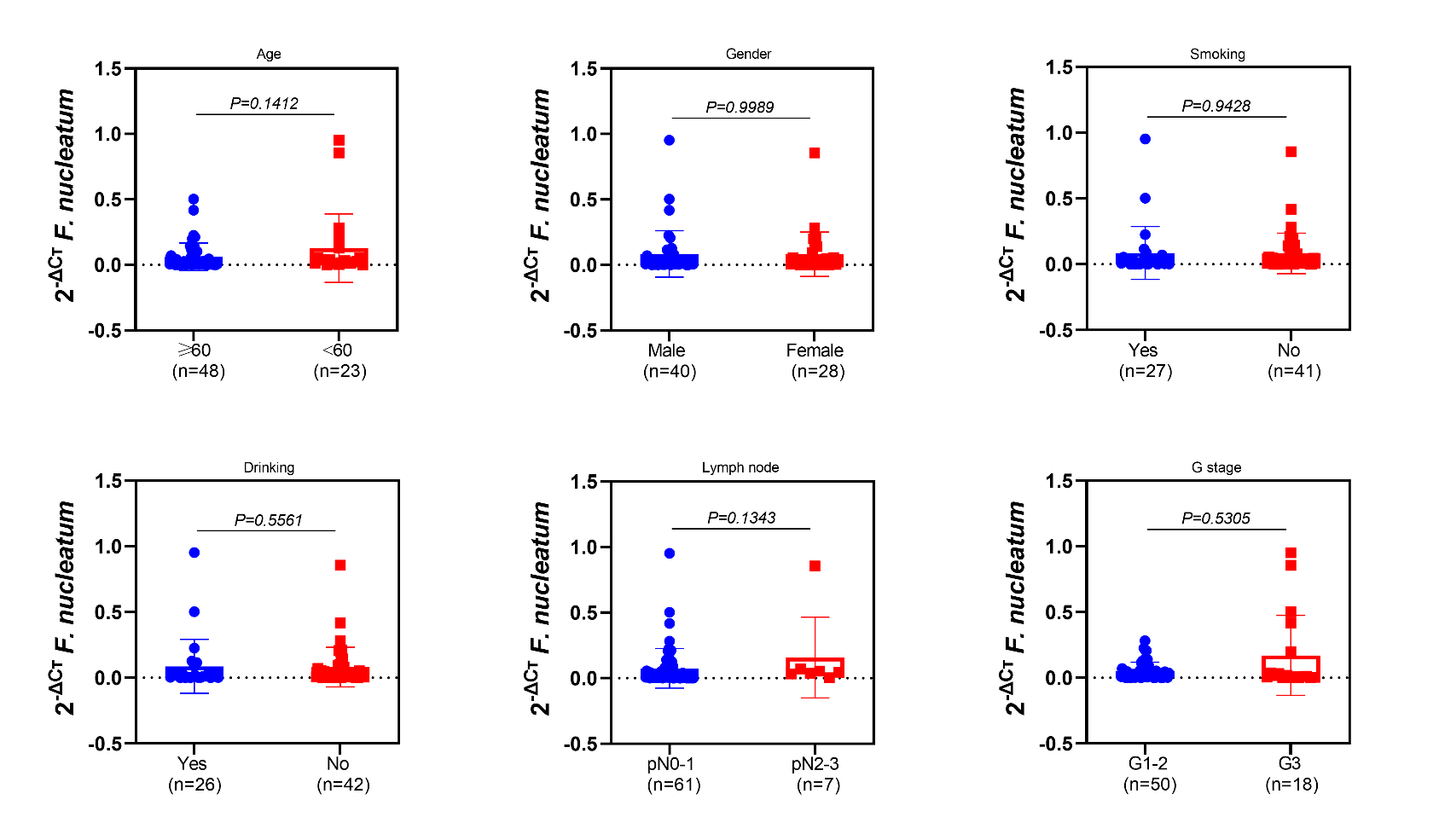


**Figure S6.** GO and Protein domain enrichment analysis of high-risk mutant genes in 7 *F. nucleatum*-negative samples. (A) GO functional enrichment analysis; (B) Protein domain enrichment analysis. The *P* value lower than 0.05 of all mutant genes in the figure.


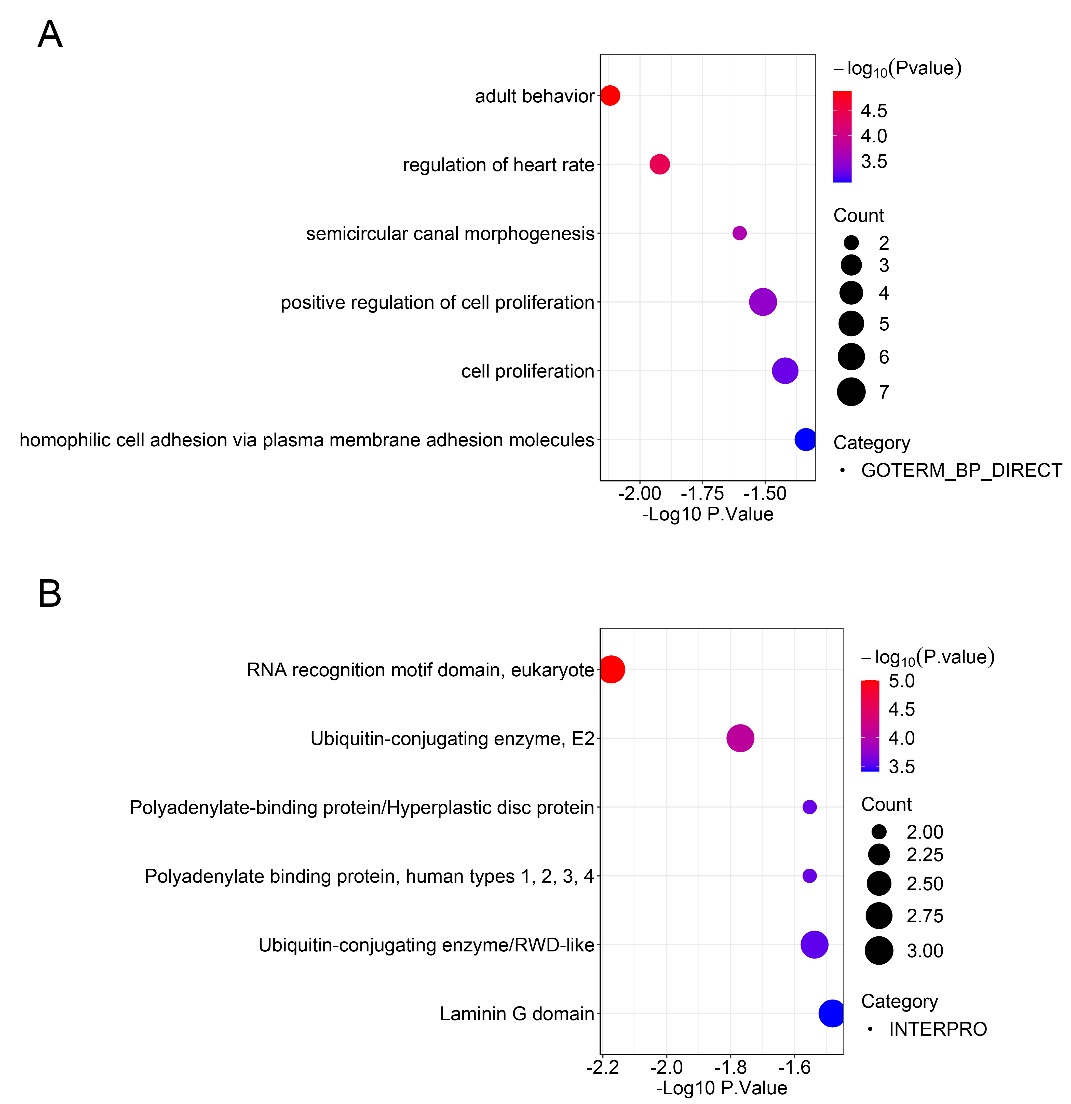

Supplement: Supplementary file 1 — Additional file 1:. [file 12866_2021_2352_MOESM1_ESM.docx]
